# Spinal Cord involvement in Dengue

**DOI:** 10.1590/0037-8682-0302-2021

**Published:** 2021-08-20

**Authors:** Laura Loeb, Paulo Afonso Mei, Fabiano Reis

**Affiliations:** 1 Faculdade de Medicina e Odontologia São Leopoldo Mandic, Campinas, SP, Brasil.; 2 Universidade Estadual de Campinas, Faculdade de Ciências Médicas, Departamento de Radiologia, Campinas, SP, Brasil.

**FIGURE 1:**
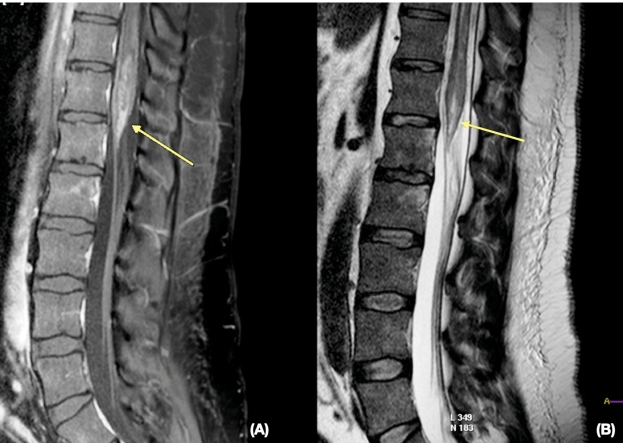
Magnetic resonance imaging of the dorsolumbar spine showing the spinal cord lesion. **(A)** Sagittal T1-weighted image, after contrast agent administration, showing moderate expansion of the distal spinal cord and conus medullaris with heterogeneous enhancement (arrow). **(B)** Sagittal T2-weighted image demonstrating a heterogeneous hyperintense lesion (arrow).

A 34-year-old male was admitted to the emergency room after developing sudden-onset paraparesis, two weeks after experiencing a febrile hemorrhagic state, accompanied by arthralgia, myalgia, and retro-orbital pain in the eyes. Dengue immunoglobulin M (IgM) serology results were positive. Other serologies for other infections were negative, including human immunodeficiency virus, zika, chikungunya, syphilis, and hepatitis C. Magnetic resonance imaging (MRI) demonstrated a spinal cord lesion consistent with transverse myelitis (TM) (Figure 1).

Cerebrospinal fluid analysis revealed normal glucose level, a protein level of 75.1 mg/dL, and a non-reagent venereal disease research laboratory (VDRL). There was partial recovery from neurological symptoms after three months following the administration of intravenous methylprednisolone and oral prednisone tapering.

Spinal cord involvement may occur between two days to more than two weeks after the first symptoms of infection^1^. However, the true prevalence of dengue-associated TM, especially postinfectious TM, is probably underestimated^2^. The immune system appears to play a role in the pathogenesis of postinfectious TM, although the presence of the virus in the spinal cord cannot be proven^2^. 

Spinal cord lesions associated with dengue should be considered in patients from endemic regions. Effective and timely diagnosis is extremely important to increase the chances of a better outcome. As shown here, MRI may be a useful diagnostic tool in patients with dengue-associated myelopathies. Nearly all cases described in the literature^3^ indicate that subsequent full neurological recovery requires intensive rehabilitation to restore muscle power.
